# Family, Domestic and Sexual Violence Presentations to a Virtual Emergency Department: A Retrospective Cohort Study

**DOI:** 10.1111/1742-6723.70316

**Published:** 2026-07-31

**Authors:** Eleanor R. Johnson, Kristina Edvardsson, Jennifer Hutton, Leesa Hooker, Brianna Pike, Bijaya Pokharel, Jessica E. Moulton, Rebecca L. Jessup

**Affiliations:** ^1^ Office of Research Northern Health Epping Victoria Australia; ^2^ School of Population and Global Health University of Melbourne Melbourne Victoria Australia; ^3^ Judith Lumley Centre, School of Nursing and Midwifery La Trobe University Bundoora Victoria Australia; ^4^ Department of General Practice, SPHERE Centre of Research Excellence in Women's Sexual and Reproductive Health in Primary Care, School of Public Health and Preventive Medicine Monash University Clayton Victoria Australia; ^5^ Victorian Virtual Emergency Department Northern Health Epping Victoria Australia; ^6^ School of Public Health La Trobe University Bundoora Victoria Australia; ^7^ School of Medicine University of Melbourne Melbourne Victoria Australia; ^8^ Reducing Gender‐Based Violence Research Group, Violet Vines Marshman Centre for Rural Health Research La Trobe Rural Health School Bendigo Victoria Australia; ^9^ School of Allied Health, Human Services and Sport La Trobe University Bundoora Victoria Australia; ^10^ Victorian Centre for Virtual Health Research Northern Health Epping Victoria Australia

**Keywords:** domestic violence, emergency medical services, family violence, intimate partner violence, reproductive coercion, sexual violence, telemedicine

## Abstract

**Background:**

Family, domestic and sexual violence (FDSV) is a global health issue affecting one in four women. Emergency departments (EDs) are often the first point of healthcare contact for people experiencing FDSV. Virtual emergency departments have been introduced in Australia to improve access to care and reduce pressure on physical EDs, but little is known about FDSV presentations in this setting. This study aimed to identify and describe FDSV presentations to the Victorian Virtual Emergency Department (VVED).

**Methods:**

A retrospective cohort study examined FDSV presentations to VVED between 1 July 2023 and 30 June 2024. Cases were identified using VVED, injury surveillance, and clinician documentation data. Records were filtered by age, sex and injury intent, followed by file review to confirm eligibility. Demographic and presentation characteristics were summarised using descriptive statistics.

**Results:**

Twenty‐one FDSV presentations were identified among 173,936 VVED presentations (0.012%). All were female, median age 37 years. Most were born in Australia (86%) and lived in socioeconomically disadvantaged areas (62%). Intimate partner violence was the most common form of FDSV (81%), and injuries above the clavicle (head, face and neck injuries) were the most frequent presentation (53%). Most patients were referred via healthcare professionals (76%). One‐third were directed to physical EDs, while most were managed virtually with follow‐up arranged through general practitioners or other healthcare providers.

**Conclusions:**

FDSV presentations were rarely identified in the virtual emergency setting, suggesting under‐detection. Virtual EDs offer opportunities to support people experiencing violence, but improved clinician training, documentation and referral pathways are needed.

## 1 Introduction

Family, domestic and sexual violence (FDSV) is a pervasive global health concern which affects one in four women [1]. FDSV disproportionately affects women compared to men regardless of sociodemographic factors and is one of the leading health risk factors for women aged 25–44 [1]. It is the leading cause of death and disability among women, with significant physical and mental health impacts, including poorer reproductive outcomes [2].

FDSV can have significant consequences for women's reproductive autonomy and sexual and reproductive health. Women who report experiences of FDSV are at an eightfold increased odds of experiencing reproductive coercion, which involves controlling decisions around pregnancy, contraception and abortion [3]. Women experiencing FDSV experience high rates of traumatic brain injury [4, 5], chronic pain, post‐traumatic stress disorder, depression and anxiety [6, 7]. Physical and sexual experiences of partner abuse have been strongly associated with a twofold increase in unintended pregnancy and abortion [3, 8]. Unsafe sex, often present in abusive relationships, is the second leading risk factor for disability adjusted life years (DALYs) in females globally [9].

In Australia, partner abuse has contributed to 41% of the homicide and violence burden in women and 18% early pregnancy loss burden [10]. One in four women report experiencing intimate partner violence in their lifetime [11], demonstrating FDSV is a significant public health issue in Australia. Women's experiences of violence annually cost the Australian and Victorian economies $26 billion and $5.3 billion, respectively [12]. Family and Domestic Violence is estimated to be more prevalent among Australian women from migrant and refugee backgrounds, with one in three reporting such experiences and coercive control, at 91%, being the most common form. Sexual violence is also a significant concern for trans and gender‐diverse people in Australia, with 61.8% of trans men and non‐binary people who were assigned female at birth reporting experiences of sexual violence or coercion in a 2018 National Survey [13].

Emergency departments (EDs) often serve as the first contact with the health system for people experiencing FDSV. ED clinicians are uniquely positioned to recognise injuries or patterns suggestive of physical abuse and provide immediate support and referral, although under‐identification remains a challenge [14, 15, 16]. Telehealth models of care, including urgent care services, have experienced rapid growth both in Australia and globally since the onset of the COVID‐19 pandemic. The Victorian Virtual Emergency Department (VVED) was established in 2020 at Northern Health in Victoria, Australia, to enhance accessibility and reduce burden on the physical ED during the pandemic and was expanded to a state‐wide service in early 2022, serving a population of over 6.5 million. Operating 24 h a day, 7 days a week, the VVED enables patients to access emergency clinicians from home or community settings, including those who do not have access to Medicare (Australia's public health insurance scheme). Since inception, the VVED has managed more than 650,000 presentations (as of September 2025). Consultations can occur via a pathway of self‐referral via nursing triage or referral by a healthcare provider to ED clinicians, for example, an ambulance paramedic or a nurse at a rural urgent care centre. The ED clinicians employed include emergency physicians, general practitioners and nurse practitioners.

VVED clinicians are in a unique position to recognise FDSV within ordinary clinical presentations. However, despite its high prevalence, FDSV screening is not routinely undertaken due to a lack of a well‐established screening and referral pathways [17, 18]. With insufficient evidence to support universal FDSV screening (i.e., asking all women) in EDs [19], a better alternative is asking only those who are at high risk for FDSV. Determining which patients are at high risk relies on clinicians' individual judgement, experience and confidence in recognising key risk factors [20]. Manually identifying high‐risk patients relies on each clinician's experience and their ability and confidence in identifying individual risk factors.

Currently, there is limited published research on FDSV presentations in the virtual ED setting, both in Australia and globally. Evidence on these presentations is important for identifying potential inequities in access and determining whether certain groups are at greater risk of under‐identification. Further, this information can help to inform clinical training and support the development of tools for FDSV identification and response in this environment. Therefore, the aim of this study was to conduct a retrospective analysis of the VVED data to identify and describe cases of FDSV in the virtual emergency environment.

## 2 Methods

### 2.1 Design

This retrospective cohort study includes FDSV presentations to the VVED over a 1‐year period from 1 July 2023 to 30 June 2024. Data sources include the Victorian Virtual Emergency database, injury surveillance data and clinician documentation.

### 2.2 Setting

The VVED is a virtually delivered public health service auspiced by Northern Health in Victoria, Australia. The service is staffed by emergency physicians, nurses and allied health professionals and offers real‐time, audio‐visual consultations, enabling clinicians to assess, diagnose, treat and escalate care as needed. People of all ages living in the state of Victoria can access the VVED either by self‐referral or through health professional referral pathways, including Ambulance Victoria, general practitioners, urgent care centres and residential aged care facilities. The service manages between 700 and 1000 presentations per day and is one of the largest virtual EDs globally.

### 2.3 Data Sources

For this study, all presentations within the specified time period were screened to identify potential cases of FDSV. While FDSV is a broad term covering many forms of abuse, our file‐review approach focused on three types of harm that could be reliably and consistently identified in clinical documentation: physical violence, sexual violence/assault and reproductive coercion. These categories were selected because they align with the injury surveillance classifications routinely collected in the service. We also note that perpetrators of FDSV may include intimate partners, family members or others; therefore, we did not restrict our screening to intimate‐partner violence alone.

To identify cases, VVED presentation and injury surveillance data for the aforementioned time period were screened. Data were filtered for age (≥ 18) and sex (female/indeterminate/other/blank) due to the high rates of FDSV in this group and because we do not routinely collect data on gender identification. Presentations were screened for the keywords ‘abuse’ or ‘assault’, and to capture FSDV presentations where this was not specified by the clinician in the primary diagnosis description (ICD‐10‐AM codes), injury surveillance records were also screened and filtered by ‘human intent’ of injury (i.e., ‘Neglect, maltreatment, assault by current or former intimate partner/other family member/other, unknown’ or ‘Sexual assault by current of former intimate partner/other family member/other, unknown’ was selected). Presentations could be either self‐disclosed or disclosed by the HCP.

File reviews were then undertaken, and if eligible to be included, data was collected in REDCap, hosted by Northern Health. All cases were de‐identified. The data collected included demographic data (sex, age, country of birth, preferred language and postcode) and presentation information (presentation, intake pathway, FDSV type, method of identification of FDSV, referrals and outcome).

### 2.4 Data Analysis

Descriptive statistics were used to summarise characteristics of cases (frequency counts, percentages, medians and ranges). Primary presentation was broadly categorised by the authors based on the clinician's documented primary presentation diagnosis in the medical record. Socio‐economic status was based on residential postcode grouped according to the Australian Bureau of Statistics Socio‐Economic Indexes for Areas (SEIFA) Index of Relative Socio‐economic Advantage and Disadvantage (IRSAD) and was derived from 2021 census data. These were determined and reported by quintiles (with 1 being most disadvantaged and 5 most advantaged).

Given the small sample size, to ensure protection of patient privacy, data were presented in aggregate form by category with proportions rounded to the nearest whole number. Where cell counts were less than five, categories were combined to minimise the risk of re‐identification.

### 2.5 Ethics

This project has received ethical approval via St Vincent's Human Research Ethics Committee (approval number 82094). This study was an audit‐level study using routinely collected data. A waiver of informed consent was granted to use the de‐identified routine clinical data.

## 3 Results

There were 173,936 VVED presentations for the one‐year time period. The demographics of the whole population sample are provided as Table 1. Following screening of VVED presentation data and injury surveillance records for the specified period, 37 presentations met the inclusion criteria for file audit and 21 of these were included in the final data set based on clinical audit. All cases were independent (no re‐presentations were identified). All included cases were de‐identified prior to analysis.

**TABLE 1:** Overall VVED population characteristics.

Characteristics	Population *n* (%)
Total presentations (excludes those who left without being seen)	173,936
*Sex*	
Female	100,883 (58%)
*Age*	
Under 18 years	57,399 (33%)
18–64 years	69,574 (40%)
65 years and over	46,963 (27%)
*Aboriginal and/or Torres Strait Islander origin*	
Yes	4348 (2%)
*Country of birth*	
Australia (inc. external territories)	158,282 (91%)
*Primary language*	
English	170,457 (98%)
*Overall referral pathway*	
Self‐referral	78,271 (45%)
Healthcare professional referral	95,665 (55%)

All identified cases were female (Table 2). The median age was 37 years (IQR 26–45), with the largest proportion aged 25–34 years (29%). Most were born in Australia (86%), with 14% born overseas. Two patients (10%) identified as Aboriginal and/or Torres Strait Islander. English was the primary language for 86%, and 14% reported a language other than English. Socioeconomic distribution based on postcode SEIFA quintile showed that most lived in the two lowest quintiles of disadvantage (62%), with only one participant (5%) residing in the most advantaged quintile.

**TABLE 2:** Demographics for family, domestic and sexual abuse presentations.

Characteristics	Sample *n* (%)^a^
*Sex*	
Female	21 (100%)
*Age*	
Median (IQR)	37 (26–45)
18–49	18 (86%)
≥ 50	3 (14%)
*Aboriginal and/or Torres Strait Islander origin*	
Yes	2 (10%)
*Country of birth*	
Australia (inc. external territories)	18 (86%)
*Primary language*	
English	18 (86%)
*Postcode SEIFA quintile*	
1—Most disadvantaged	5 (24%)
2	8 (38%)
3	4 (19%)
4	3 (14%)
5—Most advantaged	1 (5%)
*Pregnancy status*	
Pregnant	3 (14%)
*Referral pathway*	
Healthcare professional referral	16 (76%)
Ambulance Victoria	11 (52%)
Urgent care clinic	4 (19%)
Other healthcare professional	1 (5%)
Self‐referral	5 (24%)

^a^ Proportions rounded to nearest whole number.

Approximately one in four presentations was from women who self‐referred, with the remainder (76%) attending through the healthcare professional referral pathway, primarily from Ambulance Victoria (52%) (Table 2). Self‐referral was lower in the FDSV cohort than the whole of VVED population (24% vs. 45% overall). The most common presenting diagnoses were injuries above the clavicle (head, neck and face injuries) (53%), followed by cases classified as abuse (29%) and injuries below the clavicle (18%) (Table 3). Intimate partner violence was the most frequent form of FDSV (81%), with smaller proportions involving family violence (19%) and sexual violence (5%). Perpetrators were most often partners (52%) or ex‐partners (29%). In 62% of cases, FDSV was identified by referring healthcare professionals, while 38% were self‐disclosed.

**TABLE 3:** Presentation characteristics and outcomes for FDSV presentations to the VVED.

Presentation characteristics and outcomes	*n* (%)^a^
*Primary presentation diagnosis*	
Injury above clavicle (head, neck and face)	9 (53%)
Abuse^b^	5 (29%)
Injury below clavicle	3 (18%)
*FDSV type*	
Intimate partner violence	17 (81%)
Family violence	4 (19%)
Sexual violence	1 (5%)
Reproductive coercion	0
*Perpetrator*	
Partner	11 (52%)
Ex‐partner	6 (29%)
Family member	3 (14%)
Other	1 (5%)
*Method of identification of FDSV*	
Disclosed by healthcare professional with patient	13 (62%)
Self‐disclosed	8 (38%)
*Outcome*	
To attend physical ED for review	7 (33%)
To attend general practitioner for review/follow up	6 (29%)
Discharged into care of healthcare professional at scene with patient	5 (24%)
Discharged (follow up not specified)	2 (10%)
VVED follow up	1 (5%)
*Duration of consult (min)*	
Median (IQR)	55 (36–90)
Minimum	14
Maximum	289

^a^ Proportions rounded to the nearest whole number.

^b^ Abuse numbers could be recategorised into the following based on additional clinical information from notes: 1—injury below clavicle, 1—injury above clavicle, 2—injury to both above and below clavicle and 1—sexual assault.

The outcomes following presentation to the VVED varied. One in three patients was directed to attend a physical ED (33%), while others were advised to follow up with a general practitioner (29%) or were discharged into the care of another healthcare professional at the scene (24%). Two patients (10%) were discharged with no specified follow‐up, and one (5%) received follow‐up from the VVED. Consultations lasted a median of 55 min (IQR 36–90), ranging from 14 to 289 min.

## 4 Discussion

Our study identified only 21 cases of FDSV presenting to the VVED out of 173,936 total presentations during a 12‐month period, representing an extremely low presentation rate equivalent to 12 per 100,000 presentations (0.012%). Rates of FDSV presenting to Australian physical EDs among women 16–45 years have been estimated to be approximately 2% without screening, and 18% when a brief Domestic Violence Screening Tool was used [17, 21], highlighting a significant discrepancy compared to the rates identified in our study. The low rate identified is likely an underestimate of overall FDSV presenting to VVED and likely reflects more severe and physical forms of FDSV, and the challenges experienced by healthcare professionals in identifying FDSV cases in virtual consultations. The majority of identified cases involved women of reproductive age, with three pregnant women included in the study. These findings are particularly important given that young women are disproportionately represented among users of virtual EDs globally [22, 23]. Self‐referral was lower in the FDSV cohort (24% vs. 45% overall), suggesting most women experiencing FDSV were identified through healthcare professional pathways rather than through the self‐referral pathway.

Given the small number of identified FDSV cases, these findings must be interpreted with caution. Even so, several patterns appear consistent with existing evidence. Women of reproductive age represented the majority of cases, aligning with international trends [24] Although numbers were low, there was an apparent underrepresentation of women born outside Australia. This pattern should not be over‐interpreted, but it may reflect broader inequities that we have found in other research we have conducted in the VVED (unpublished), including language‐related and structural access barriers that can disproportionately affect culturally and racially marginalised women [25]. While virtual health can reduce some of these barriers, it may create new ones for others, including culturally and racially marginalised women, those with limited digital literacy, women with disability and those living in rural or remote regions with inadequate digital infrastructure [26]. These intersecting disadvantages highlight that virtual care must be designed in a way that addresses equity of access. This is supported by a global review we have recently conducted on the use of virtual EDs conducted by the authors, which indicates that non‐English‐speaking individuals and those from lower socioeconomic backgrounds are underrepresented among users (unpublished) [27].

The low rate of identified FDSV cases may reflect not only under‐recognition by clinicians but also barriers to disclosure that are specific to the virtual consultation environment. Even when a patient experiencing FDSV accesses the VVED, the consultation itself may not constitute a safe moment for disclosure. A perpetrator or other family member may be present in the room during the call, and patients may have limited ability to signal this to the clinician. Coercive control can further restrict a patient's capacity to speak freely, even when physically alone, through fear of retaliation if the contact is later discovered. Shame and self‐blame remain significant barriers to disclosure in any emergency setting [16]. Clinicians working in virtual environments cannot rely on the physical cues (e.g., hesitation, affect or injury patterns inconsistent with the reported mechanism) that might prompt further enquiry in a face‐to‐face consultation.

Aboriginal and Torres Strait Islander women were overrepresented in our sample, consistent with research demonstrating their disproportionate exposure to violence and the high likelihood that incidents go unreported [28]. Interestingly, Aboriginal and Torres Strait Islander people are over‐represented in the VVED as a whole (representing 2% of all presentations, against a statewide population of around 1% [29]). Presentations were also concentrated in more disadvantaged areas, which aligns with well‐established associations between socioeconomic disadvantage and increased risk, frequency and severity of FDSV [30]. Pregnancy emerged as a period of heightened vulnerability, echoing research indicating that violence may begin or escalate during pregnancy and that pregnant women may seek care due to concerns about their own and their unborn child's safety [31].

Delivering FDSV care in the virtual emergency setting presents unique benefits and challenges. Intimate partner violence was the most common form of violence identified in our study, and partners or ex‐partners were the most frequent perpetrators, highlighting the ongoing risks women face whether remaining in or leaving abusive relationships [32, 33]. Importantly, no cases of reproductive coercion were identified in our dataset. This may reflect a lack of awareness among health professionals and established definitions of this form of violence, and the associated challenges of recognising and documenting this form of violence in the virtual setting. Most referrals came via the healthcare professional pathway, with the VVED primarily used to assess injury severity and the need for escalation. Around one‐third of cases were referred to physical EDs, but most were managed virtually, with follow‐up arranged via general practitioners or other healthcare professionals. This demonstrates the VVED's potential to reduce burden on physical EDs while also minimising travel and waiting times for patients—an important consideration for those already experiencing barriers to safety and care. The median consult time of 55 min reflects the complexity of FDSV presentations and the extended clinician time required for assessment, safety planning and organisation of referrals.

Identification and management of FDSV in the virtual ED environment presents a new opportunity to support those at risk, but it requires whole of systems support including staff training, guidelines, leadership and policy [20, 34]. Clinicians need to balance the acute clinical assessment with both trauma‐informed inquiry and a sensitive approach to potential violence, often under conditions where privacy and patient safety are uncertain [35]. This study suggests that, similar to what has been found in face‐to‐face EDs, there is a need to enhance clinician training to improve recognition of subtle signs of FDSV and to adapt safety planning to the virtual context [35]. The low number of identified cases, despite the large volume of virtual presentations, points to under‐detection likely related to limited healthcare provider training, the absence of tailored protocols, limitations in documentation and coding and challenges in establishing rapport and ensuring confidentiality in virtual consultations. Strengthening case finding through staff training and embedding prompts in clinical documentation could help improve identification and surveillance of FDSV cases in this setting.

While physical EDs have well established protocols for identifying and responding to FDSV, the virtual emergency setting introduces unique dynamics which makes caring for people experiencing FDSV potentially both easier in some contexts and more challenging in others. The service reduces barriers to care in terms of removing the need to travel long distances, being accessible outside of standard operating hours and being able to seek advice discreetly when under surveillance, meaning people experiencing FDSV may intentionally seek this service. In addition, it reduces the distress that can occur from attending and waiting in a busy ED [36]. However, the virtual setting also means clinicians may find it difficult to interpret and respond to non‐verbal cues. Asking patients direct questions about FDSV exposure is also more challenging as providers often cannot be certain of patient privacy, and the risk that a perpetrator is potentially present in the background. A further consideration specific to virtual consultations is the risk of device surveillance. A perpetrator may monitor a patient's phone, tablet or computer and may be able to review call history, browser activity or messaging, potentially exposing both the patient's attempt to seek help and any safety planning discussed during the consultation. Building the trust necessary for FDSV disclosure in a virtual encounter requires deliberate communication strategies, including explicitly offering privacy (e.g., asking whether the patient is alone and able to speak freely), using trauma‐informed language and clearly explaining confidentiality, its limits and any mandatory reporting obligations before asking about violence exposure.

The strength of this study lies in its population approach, drawing on 12 months of complete data from a state‐wide virtual ED in Australia that is now one of the largest in the world. However, several limitations should be considered when interpreting the findings. FDSV is likely under‐detected due to reliance on routine clinical documentation and coding, and the small number of identified cases limits the ability to draw strong conclusions about demographic patterns or broader prevalence. The lack of disclosure by patients and the methods used to identify FDSV are also limitations and likely resulted in under‐identification of cases. Recognising and responding to sexual assault in a virtual environment presents inherent challenges, as physical examination, forensic evidence collection and immediate access to specialist support are not possible in the same way as in traditional EDs, and this may have also limited the documentation of cases. As this model of care evolves, improved workflows and clearer pathways for responding to sexual assault presentations may improve data capture. Use of routinely collected data also meant that information on sexual orientation and gender identity was unavailable. Gender was captured using sex assigned at registration (male, female, other), which may not reflect the experiences of transgender and gender‐diverse people. Given that LGBTQI+ communities experience intimate partner violence at higher rates than heterosexual populations [37], the absence of these data limits understanding of their experiences in virtual ED care. Future changes to minimum datasets collected during triage, including standardised collection of gender identity and sexual orientation, would improve identification of this vulnerable community. Finally, the study design did not allow for exploration of patient or clinician perspectives, which may provide a deeper understanding of barriers and facilitators for FDSV identification in the virtual emergency context.

## 5 Conclusion

Our audit identified very few FDSV presentations to the VVED, suggesting under‐detection in the virtual environment. Although numbers were small, the patterns observed were consistent with known FDSV risk groups. Virtual EDs offer new opportunities to support people experiencing violence, but effective identification requires clearer pathways, strengthened clinician training and systems that support the unique challenges of virtual care. Further research using a co‐design approach would likely ensure the creation of virtual emergency models that are safe, equitable and responsive for all FDSV survivors.

## Author Contributions

All authors contributed to the conception and design of the study. E.R.J. executed the study and analysis with assistance from J.H. and R.L.J. All authors provided input regarding the interpretation of results and approved the final manuscript.

## Funding

This paper is supported by a La Trobe University ‘Healthy people, families and communities’ themed 2024 Research Capability Grant.

## Conflicts of Interest

The authors declare no conflicts of interest.

## Data Availability

The data used in this study was accessed from the Victorian Virtual Emergency database. Access to this database can be discussed with the data custodian.
